# The role of the mean platelet volume and neutrophil-to-lymphocyte ratio in peritonsillar abscesses^☆^^☆☆^

**DOI:** 10.1016/j.bjorl.2015.11.018

**Published:** 2016-03-28

**Authors:** Mehmet Şentürk, İsa Azgın, Gültekin Övet, Necat Alataş, Betül Ağırgöl, Esra Yılmaz

**Affiliations:** Konya Education and Research Hospital, Department of Otolaryngology Head and Neck Surgery, Konya, Turkey

**Keywords:** Mean platelet volume, Neutrophil-to-lymphcyte rate, Peritonsillar abscess, Treatment, Volume plaquetário médio, Relação neutrófilos/linfócitos, Abscesso periamigdaliano, Tratamento

## Abstract

**Introduction:**

Peritonsillar abscess is a serious infectious disease of the tonsillar tissue. Treatment generally requires both medical and surgical approaches to relieve the symptoms. Recently, in addition to clinical follow-up, some inflammatory markers, such as the mean platelet volume and neutrophil-to-lymphocyte ratio, have been considered to be additional inflammatory monitoring markers in inflammatory diseases.

**Objective:**

The aim of this study was to describe the role of mean platelet volume and neutrophil-to-lymphocyte ratio in patients with peritonsillar abscess.

**Methods:**

A retrospective study was conducted in 88 patients with peritonsillar abscess and 88 healthy individuals. We analyzed the white blood cell count, neutrophil count, lymphocyte count, platelet count, C-reactive protein, mean platelet volume and neutrophil-to-lymphocyte ratio values and compared them among the patient and control groups.

**Results:**

The mean platelet volume levels were significantly higher in the peritonsillar abscess pretreatment group than in the peritonsillar abscess posttreatment group and the control group. A mean platelet volume value of 8.7 was the optimal cut-off value for evaluating the sensitivity, specificity, positive predictive value and negative predictive value of 75%, 65.9%, 68% and 72%, respectively. The neutrophil-to-lymphocyte ratio levels were significantly higher in the peritonsillar abscess pretreatment group than in the peritonsillar abscess post-treatment group and the control group. A neutrophil-to-lymphocyte ratio value of 3.08 was the optimal cut-off value for evaluating the sensitivity, specificity, positive predictive value and negative predictive value of 90.9%, 90.9%, 90.9% and 90.9%, respectively. While the white blood cell count, neutrophil count, lymphocyte count and C-reactive protein values were significantly different among the patient and control groups (*p* < 0.05), the platelet count was not significantly different among the patient and control groups (*p* > 0.05).

**Conclusion:**

The mean platelet volume and neutrophil-to-lymphocyte ratio values made us think that these parameters were quick, inexpensive and reliable inflammatory follow-up parameters and could be easily integrated into daily practice for peritonsillar abscess treatment except platelet count.

## Resumo

**Introdução:**

O abscesso periamigdaliano (APA) é uma doença infecciosa grave do tecido tonsilar. O seu tratamento geralmente requer uma abordagem medicamentosa e cirúrgica para o alívio dos sintomas. Recentemente, além do acompanhamento clínico, alguns marcadores inflamatórios, como o volume plaquetário médio (VPM) e a relação neutrófilos/linfócitos (RN/L), foram considerados marcadores de monitoramento adicionais em doenças inflamatórias.

**Objetivo:**

O objetivo deste estudo foi descrever o papel os VPM e a RN/L em pacientes com APA.

**Método:**

Estudo retrospectivo realizado com 88 pacientes com ATP e 88 indivíduos saudáveis. Analisamos a contagem de leucócitos, neutrófilos, linfócitos, plaquetas, proteína C-reativa (PCR), VPM e RN/L e a comparamos os valores entre o grupo de pacientes e grupo controle.

**Resultados:**

Os níveis de VPM eram significativamente maiores no grupo APA pré-tratamento que no grupo APA pós-tratamento e no grupo controle. Um valor de corte de 8,7 para o VPM foi considerado ideal para avaliar sensibilidade, especificidade, valor preditivo positivo e valor preditivo negativo de 75, 65,9, 68 e 72%, respectivamente. Os níveis da RN/L eram significantemente maiores no grupo APA pré-tratamento que no grupo APA pós-tratamento e no grupo controle. Um valor de 3,08 para a RN/L foi o valor de corte ideal para avaliar sensibilidade, especificidade, valor preditivo positivo e valor preditivo negativo de 90,9, 90,9, 90,9 e 90,9%, respectivamente. Enquanto a contagem dos valores de leucócitos, neutrófilos, linfócitos e PCR foi significantemente diferente entre os grupos de pacientes e controle (p<0,05), a contagem de plaquetas não foi (p>0,05).

**Conclusão:**

Os valores de VPM e RN/L sugerem que estes são parâmetros inflamatórios de acompanhamento rápido, barato e confiável, e que podem ser facilmente integrados à prática diária para o tratamento de APA, exceto pela contagem de plaquetas.

## Introduction

Peritonsillar abscess is one of the most common deep neck infections and is characterized by the accumulation of pus between the palatine tonsils and the superior pharyngeal constrictor muscle.^1^, ^2^ Airway obstruction, abscess rupture, pus aspiration, asphyxia and septicemia may develop as a result of inadequate treatment or a progressed infection.^3^

This severe infection needs to be followed-up clinically as well as by performing a hematological parameter follow-up.^2^ Although procalcitonin, pro-adrenomedullin,^4^ serum amyloid A, fibrinogen and CD-14 binding protein^5^ are among the sensitive follow-up parameters that are used for following up on inflammation, they may not be available in every inpatient service's laboratories and present an additional cost. However, simple hemogram values, such as the white blood cell (WBC) count, neutrophil count and lymphocyte count, which are among the most commonly used parameters, are often available in every clinical laboratory and bear no additional cost. A routine pus culture rarely changes the course of treatment; however, it is expensive and should not be sent to the laboratory unless there is a finding that requires a re-assessment of the clinical results.^2^ Moreover, at least 48 h are necessary to receive results from abscess cultures. Delays in treatment selection may suppress the improvement in symptoms and can lead to an increase in the spread of infection and loss of patients’ time.^6^

MPV is one of the platelet function indicators that reflects the platelet production rate and stimulation.^7^ It was found that the MPV, which is available to be measured in routine hematological examinations, directly correlates with the course of the disease, such as sepsis in neonates with a very low birth weight,^8^ pediatric acute pyelonephritis^9^ and gastric cancer.^10^

The use of the neutrophil-to-lymphocyte ratio (NLR), which is obtained by dividing the neutrophil count by the number of lymphocytes, was suggested by Zahorec,^5^ and it was concluded that it is a significant prognostic parameter for patients undergoing treatment in the intensive care unit. NLR was also reported as one of the most reliable indicators in distinguishing between patients with or without bloodstream infections for those admitted to emergency services with a suspected bloodstream infection^11^; as a better follow-up parameter than the CRP levels, WBC count and neutrophil count for the follow-up of bacteriemia^4^; and as a prognostic factor in phase I–III patients with colorectal cancer after curative surgery.^12^

Although they were reported to be useful, inexpensive, effective, reliable and easily accessible routine hemogram parameters for follow-up in many inflammatory diseases, we could not find any study in the literature regarding the use of MPV and NLR in PTA treatment. The aim of this study is to evaluate the effectiveness and usefulness of MPV and NLR levels in addition to the routine hematological inflammatory follow-up parameters.

## Methods

Retrospectively, 88 patients who had been hospitalized because of PTA between January 2008 and February 2015 and 88 healthy individuals with normal hematological parameters were included in the study. Patients with PTA were selected from patients who were diagnosed with PTA by an 18 gauge needle aspiration that was performed on the junction of the upper pole of the medial swelled tonsils and uvula base and/or from patients who had pus draining from the incision. The protocol of this study was approved by the Institutional Ethical Committee (Decision n° 2015/123).

The clinical records of patients in the experimental and control groups were screened, and patient information regarding age, gender, clinical history and course of the disease were examined. Patients with hypertension, diabetes mellitus, metabolic syndrome, coronary heart disease, thyroid dysfunction, renal and hepatic dysfunction, malignancy, surgical history in the last 3 months, deviation of the nasal septum, systemic inflammatory disease, anemia, and chronic obstructive pulmonary disease (COPD) as well as any patients who smoked or used medication for chronic inflammation were excluded from the study.

The patients were divided into (i) pre-treatment, (ii) post-treatment, and (iii) control groups. In the patient group, the leukocyte count (WBC), neutrophil count, lymphocyte count, platelet count, C-reactive protein (CRP) levels, MPV levels and NLR levels were examined both at the time of hospitalization (pre-treatment) and before discharge (post-treatment). Although the MPV values are automatically calculated by devices in routine hemogram parameters, the NLR values were obtained by dividing the neutrophil count by the lymphocyte count. For healthy individuals in the control group, the other parameters that were mentioned above were examined, except for CRP (because CRP is an acute phase reactant that indicates acute inflammation).

Blood samples were collected in tubes containing ethylene diaminetetraacetic acid (EDTA) and were analyzed in our laboratory. The hematological parameters of patients were analyzed with a Sysmex XE-2100T Automated Hematology System (Japan), and nephelometric analysis was performed with a Siemens BNTM-II, Automated Analyser (Germany). The reference range in our laboratory was 6.5–12 fL for MPV, 4.4–11.3 (103 μL) for leukocytes, 150–450 (103 μL) for platelets, 0.9–3.2 (103 μL) for lymphocytes and 0–5 mg/L for CRP.

### Statistical analysis

All of the statistical calculations were performed using the SPSS statistical software package (SPSS, version 16.0 for Windows, SPSS, Inc., Chicago, IL). The suitability of the variables for a normal distribution was tested by the Kolmogorov–Smirnov test. The mean ± standard deviation (SD) of the data was given as descriptive statistics. The variables in the normal distribution were compared using a *t*-test. The Mann–Whitney *U* test was used for the variables that did not fit a normal distribution. ROC analysis was performed for the MPV and NLR to assess the sensitivity, specificity, positive predictive value and negative predictive value. A probability value of *p* < 0.05 was considered statistically significant.

## Results

There were 88 patients in the PTA group; 46 were male (52.3%) and 42 were female (47.7%). The mean age of the patient group was 35.90 ± 11.62 years. In the control group, there were 88 healthy subjects; 47 were male (53.4%) and 41 were female (46.6%). The mean age of the control group was 34.59 ± 12.84. There was no significant difference among patient and control groups in terms of age (*p* = 0.480) or gender (*p* = 1.000).

**Figure 1:**
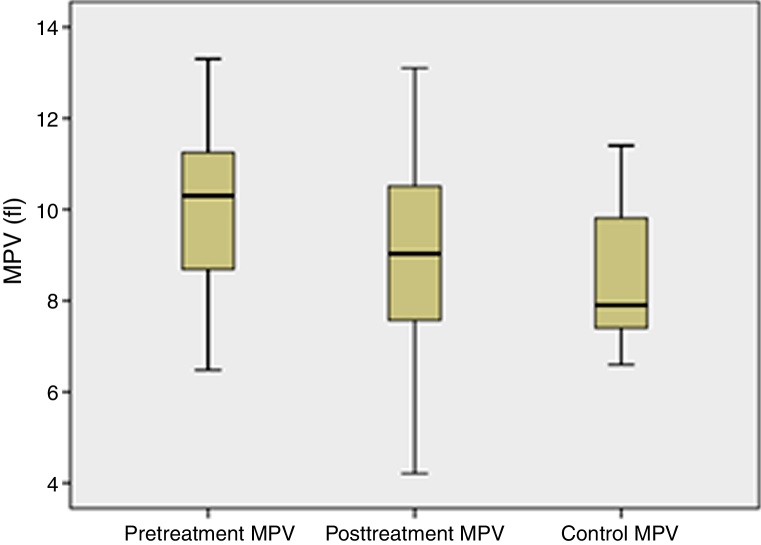
MPV values in the PTA pretreatment and post-treatment groups and the control group.

Considering the MPV values of the groups, the pre-treatment MPV values of the PTA group had higher MPV levels than the PTA post-treatment group (10.03 ± 1.56 vs. 8.76 ± 2.12 fL; *p* = 0.001) and the control group (10.03 ± 1.56 vs. 8.45 ± 1.35 fL; *p* = 0.001); the differences were statistically significant. ROC curve analysis revealed that the MPV level cut-off point for a diagnosis of PTA was 8.7 fL, with a sensitivity, specificity, positive predictive value (PPV), and negative predictive value (NPV) of 75%, 65.9%, 68% and 72%, respectively (area under curve: 0.782). The MPV values of the groups are depicted in Fig. 1.

**Figure 2:**
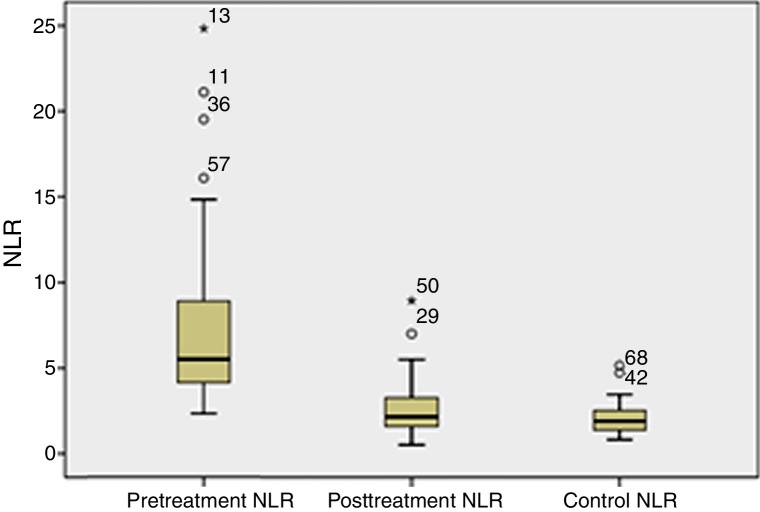
NLR values in the PTA pretreatment and post-treatment groups and the control group.

The pre-treatment NLR values of the PTA group had higher levels of NLR than the PTA post-treatment group (7.12 ± 4.29 vs. 2.60 ± 1.44; *p* = 0.001) and the control groups (7.12 ± 4.29 vs. 2.02 ± 0.80; *p* = 0.001); the differences were statistically significant. ROC curve analysis revealed that the NLR level cut-off point for a diagnosis of PTA was 3.08, with a sensitivity, specificity, positive predictive value (PPV), and negative predictive value (NPV) of 90.9%, 90.9%, 90.9% and 90.9%, respectively (area under curve: 0.976). The NLR values of the groups are depicted in Fig. 2.

The mean values of the white blood cell count, neutrophil count and lymphocyte count were significantly different among patient and control groups (*p* < 0.05). However, the platelet count was not statistically significantly different among patient and control groups (*p* > 0.05) (Table 1).

**Table 1:** Hemogram test results and the MPV, NLR and PLR values of the pre-treatment and post-treatment patients and the controls.

Parameter	Group 1 (Pre-treatment group of PTA)	Group 2 (Post-treatment group of PTA)	Group 3 (Control group)	*p*-Value
WBC (10^3^/μL)	15.13 ± 4.19	7.65 ± 1.72	7.18 ± 1.43	0.001^a^, ^b^; 0.51^c^
NEU (10^3^/μL)	11.82 ± 3.98	4.61 ± 1.20	4.19 ± 1.13	0.001^a^, ^b^; 0.18^c^
LYM (10^3^/μL)	1.96 ± 0.76	2.08 ± 0.83	2.22 ± 0.56	0.291^a^; 0.012^b^; 0.191^c^
PLT (10^3^/μL)	273.65 ± 87.39	273.58 ± 59.24	258.03 ± 62.90	0.993^a^; 0.176^b^; 0.93^c^
CRP (mg/L)	116.34 ± 73.22	3.09 ± 1.03	NS	0.001^a^; NS^b^, ^c^
MPV (fL)	10.03 ± 1.56	8.76 ± 2.12	8.45 ± 1.35	0,001^a^, ^b^, 0.247^c^
NLR	7.12 ± 4.29	2.60 ± 1.44	2.02 ± 0.80	0.001^a^, ^b^; 0.006^c^

NS, non studied.

a Group 1 vs. Group 2.

b Group 1 vs. Group 3.

c Group 2 vs. Group 3.

## Discussion

Peritonsillar abscesses have been well described; the disease was discussed in two recent reviews from various aspects, such as differentiation from infective mononucleosis, pus culture features, imaging modalities, surgical treatment approaches, medical treatment features, admission or outpatient management and interval tonsillectomy issues.^2^, ^3^ However, follow-up with additional inflammatory markers was not discussed in these reviews. To the best of our knowledge, there is no publication regarding the investigation of the WBC count, lymphocyte count, platelet count and CRP levels together with the MPV and NLR levels. Thus, our study was intended to compare these parameters between patients with peritonsillar abscesses and a control group.

In the medical and surgical treatment of patients with a peritonsillar abscess, the hematological infection follow-up parameters are required as well as a routine follow-up for the improvement in symptoms. The white blood cell count (WBC), neutrophil count, lymphocyte count and C-reactive protein (CRP) levels are among the most commonly used hematological parameters for monitoring the effectiveness of infectious disease treatment.^13^ In the pre-treatment and post-treatment comparison of the white blood cell count (WBC), neutrophil count, lymphocyte count, platelet count and CRP levels of patients with PTA, the difference between the pre- and post-treatment WBC count, neutrophil count and CRP levels of patients with PTA were statistically significant, whereas there was no statistically significant difference between the lymphocyte count and platelet count in our study.

The conditions that cause an inflammatory response are characterized by the recognition of damaged areas by inflammatory cells, specific accumulation of leukocyte subsets and elimination of the hostile agent. Systemic bacterial inflammation and sepsis are characterized by a decrease in the number of lymphocytes and an increase in the number of neutrophils. It was reported that the mechanism responsible for lymphopenia is related to the margination and redistribution of lymphocytes in the lymphatic system, whereas accumulation of neutrophils at the site, late apoptosis and stem cell stimulation were reported to be responsible for neutrophilia. The inflammatory/immune response against stress can be effectively revealed by NLR, which is the ratio of the neutrophil count to the lymphocyte count.^5^ The NLR was shown to be a biomarker that differentiates bacteremia in patients from a suspected community-acquired infection.^11^ It was shown that the NLR values differentiate bacteremia significantly better in patients admitted to the emergency department than the routine parameters of the CRP level, WBC count and neutrophil count.^4^ In addition, NLR was reported to be superior to other routine hemogram values in distinguishing community-acquired pneumonia and pulmonary tuberculosis.^14^ NLR was also reported to be a very effective parameter that provides information about the prognosis and follow-up of myocardial infarction,^15^ gangrenous appendicitis^16^ and colorectal cancer.^12^ According to our study, there was a statistically significant decrease in post-treatment NLR values compared to the pre-treatment ones.

Platelets are involved in the pathogenesis of infectious diseases in addition to their primary hemostatic functions.^17^ There can be changes in the platelet diameter in response to infection during the course of infectious diseases; there may be an increase in MPV during a serious infection, and this increase in MPV may be a result of the rapid release of platelets in the spleen.^18^ In other words, MPV increases in the initial phase of infections as an inflammatory marker. Additionally, it was found to be increased compared to the controls in acute pyelonephritis,^9^ in acidic fluid infections,^19^ in severe community-acquired pneumonia that requires hospitalization^20^ and in infective endocarditis.^21^ In our study, it was observed that the MPV parameter in patients with peritonsillar abscesses was increased in the pre-treatment phase and significantly decreased after treatment.

## Conclusion

In our study, it was believed that the MPV and NLR values could be used as a quick, inexpensive and reliable inflammatory follow-up parameter during PTA treatment and could easily be integrated into the daily practice for PTA treatment. It was also observed that platelet count is not affected by the course of PTA. In addition, this study is the first investigation of the effectiveness and significance of the MPV and NLR values in PTA's clinical follow-up. Regarding this issue, further studies are needed to assess changes in inflammatory markers in larger groups of patients with PTA.

## Conflicts of interest

The authors declare no conflicts of interest.
